# Supplementary material for: Image processing tools for petabyte-scale light sheet microscopy data
Source: Nat Methods. 2024 Oct 17;21(12):2342–52. doi: 10.1038/s41592-024-02475-4 (PMC11621031; doi:10.1038/s41592-024-02475-4)
Supplement: Supplementary file 2 — Reporting Summary [file 41592_2024_2475_MOESM2_ESM.pdf]

nature portfolio

Corresponding author(s): Eric Betzig, Srigokul Upadhyayula, Xiongtao Ruan

Last updated by author(s): Sep 8, 2024

Reporting Summary

Nature Portfolio wishes to improve the reproducibility of the work that we publish. This form provides structure for consistency and transparency in reporting. For further information on Nature Portfolio policies, see our Editorial Policies and the Editorial Policy Checklist.

Statistics

For all statistical analyses, confirm that the following items are present in the figure legend, table legend, main text, or Methods section.

n/a	Confirmed	
☐	☒	The exact sample size (*n*) for each experimental group/condition, given as a discrete number and unit of measurement
☐	☒	A statement on whether measurements were taken from distinct samples or whether the same sample was measured repeatedly
☒	☐	The statistical test(s) used AND whether they are one- or two-sided<br>*Only common tests should be described solely by name; describe more complex techniques in the Methods section.*
☒	☐	A description of all covariates tested
☒	☐	A description of any assumptions or corrections, such as tests of normality and adjustment for multiple comparisons
☒	☐	A full description of the statistical parameters including central tendency (e.g. means) or other basic estimates (e.g. regression coefficient) AND variation (e.g. standard deviation) or associated estimates of uncertainty (e.g. confidence intervals)
☐	☒	For null hypothesis testing, the test statistic (e.g. *F*, *t*, *r*) with confidence intervals, effect sizes, degrees of freedom and *P* value noted<br>*Give P values as exact values whenever suitable.*
☒	☐	For Bayesian analysis, information on the choice of priors and Markov chain Monte Carlo settings
☒	☐	For hierarchical and complex designs, identification of the appropriate level for tests and full reporting of outcomes
☒	☐	Estimates of effect sizes (e.g. Cohen's *d*, Pearson's *r*), indicating how they were calculated

Our web collection on statistics for biologists contains articles on many of the points above.

Software and code

Policy information about availability of computer code

Data collection	LabView and Matlab
Data analysis	https://github.com/abcucberkeley/PetaKit5D<br>Matlab (R2023a, MathWorks), Python (3.8.8), Imaris (10.0, Oxford Instruments), Amira (2023.2, Thermo Fisher), Fiji (1.53t), and NVIDIA IndeX (NVIDIA), BaSiC (https://github.com/marrlab/BaSiC).

For manuscripts utilizing custom algorithms or software that are central to the research but not yet described in published literature, software must be made available to editors and reviewers. We strongly encourage code deposition in a community repository (e.g. GitHub). See the Nature Portfolio guidelines for submitting code & software for further information.

Data

Policy information about availability of data

All manuscripts must include a data availability statement. This statement should provide the following information, where applicable:

- Accession codes, unique identifiers, or web links for publicly available datasets
- A description of any restrictions on data availability
- For clinical datasets or third party data, please ensure that the statement adheres to our policy

Data will be made available upon reasonable request and the means of transfer provided (disks, globus, etc). Since the datasets are large (e.g., the VNC data 38 TiB and time-lapse live cell data 8.1 TiB) for any open repository, we uploaded representative subsets of the datasets to Dryad, and they can be accessed from: https://

doi.org/10.5061/dryad.kh18932g4 (time-lapse live cell imaging data, 2-photon live mouse brain imaging data, oblique illumination "phase" imaging of HeLa cells, widefield and confocal imaging data) and https://doi.org/10.5061/dryad.jq2bvq8jd (VNC data).

The cell data for deconvolution comparison for light sheet microscopy data is from Liu et al., Science Advances, 2023, and can be accessed from: https://doi.org/10.6078/D1VT6K , https://doi.org/10.6078/D1MB09 , and https://doi.org/10.6078/D1GM7G .

The stitching comparison dataset (ExA-SPIM) is from Glaser et al., eLife, 2023, and can be accessed from: s3://aind-open-data/exaSPIM_615296_2022-09-28_11-47-06 using AWS CLI (https://github.com/aws/aws-cli) or following the instructions at: https://allenneuraldynamics. github.io/data.html .

We also prepared two demo datasets for users to test the software: https://zenodo.org/records/10471979 (for light sheet microscopy data) and https://zenodo.org/records/11500863 (for non-light sheet microscopy data: 2-photon, confocal, phase and widefield).

Human research participants

Policy information about studies involving human research participants and Sex and Gender in Research.

Reporting on sex and gender	N/A
Population characteristics	N/A
Recruitment	N/A
Ethics oversight	N/A

Note that full information on the approval of the study protocol must also be provided in the manuscript.

Field-specific reporting

Please select the one below that is the best fit for your research. If you are not sure, read the appropriate sections before making your selection.

- [x] Life sciences
- [ ] Behavioural & social sciences
- [ ] Ecological, evolutionary & environmental sciences

For a reference copy of the document with all sections, see nature.com/documents/nr-reporting-summary-flat.pdf

Life sciences study design

All studies must disclose on these points even when the disclosure is negative.

Sample size	No sample-size calculations were needed in this study. We used real or synthetic image data to run the computational benchmarks to demonstrate the performance of our software.
Data exclusions	No data were excluded in the manuscript.
Replication	No replicate imaging experiments were performed. For computational benchmarks, all replicates (typically 3-10) were performed independently and successful. The replication numbers are described in the manuscript.
Randomization	There were no experimental groups, so randomization was not applicable.
Blinding	There were no experimental groups, so blinding was not applicable.

Reporting for specific materials, systems and methods

We require information from authors about some types of materials, experimental systems and methods used in many studies. Here, indicate whether each material, system or method listed is relevant to your study. If you are not sure if a list item applies to your research, read the appropriate section before selecting a response.

Materials & experimental systems

n/a	Involved in the study
☐	☒ Antibodies
☐	☒ Eukaryotic cell lines
☒	☐ Palaeontology and archaeology
☐	☒ Animals and other organisms
☒	☐ Clinical data
☒	☐ Dual use research of concern

Methods

n/a	Involved in the study
☒	☐ ChIP-seq
☒	☐ Flow cytometry
☒	☐ MRI-based neuroimaging

Antibodies

Antibodies used	Chicken anti-GFP (1:1000, Abcam, ab13970)<br>Rabbit anti-dsRed (1:1000, Takara Bio, 632496)<br>Goat anti-chicken IgY Alexa Fluor 488 (1:500, Invitrogen, A11039)<br>Goat anti-rabbit IgG Alexa Fluor 568 (1:500, Invitrogen, A11011)
Validation	Chicken anti-GFP: specific for GFP, validated for immunofluorescence from manufacturer: https://www.abcam.com/en-us/products/primary-antibodies/gfp-antibody-ab13970<br>Rabbit anti-dsRed: specific for dsRed, extensively validated for immunofluorescence with the summary and publications: https://www.takarabio.com/learning-centers/gene-function/fluorescent-proteins/fluorescent-protein-antibody-citations/rfp-antibody-citations

Eukaryotic cell lines

Policy information about cell lines and Sex and Gender in Research

Cell line source(s)	LLC-PK1 cells were gifts from M. Davidson at Florida State University, originally obtained from ATCC.<br>HeLa (CCL-2) cells were purchased from ATCC.
Authentication	No further authentication was performed for this study.
Mycoplasma contamination	The cell lines were tested for mycoplasma contamination and the results were negative.
Commonly misidentified lines<br>(See ICLAC register)	None of the cell lines used here belongs to commonly misidentified lines.

Animals and other research organisms

Policy information about studies involving animals; ARRIVE guidelines recommended for reporting animal research, and Sex and Gender in Research

Laboratory animals	Transgenic Thy1-YFP-H mice (The Jackson Laboratory) of 8 weeks or older; adult (female, 4-6 days after eclosure) fruit flies (Drosophila melanogaster).
Wild animals	No wild animals were used in this study.
Reporting on sex	Male or female mice; female fruit flies.
Field-collected samples	No field-collected samples were used in this study.
Ethics oversight	All mice experiments were conducted at Janelia Research Campus, Howard Hughes Medical Institute (HHMI) in accordance with the US National Institutes of Health Guide for the Care and Use of Laboratory Animals. Procedures and protocols were approved by the Institutional Animal Care and Use Committee of the Janelia Research Campus, HHMI.

Note that full information on the approval of the study protocol must also be provided in the manuscript.
